# Speckle tracking echocardiographic deformation indices in Chagas and idiopathic dilated cardiomyopathy: Incremental prognostic value of longitudinal strain

**DOI:** 10.1371/journal.pone.0221028

**Published:** 2019-08-22

**Authors:** Omar Ribeiro Santos Junior, Manoel Otávio da Costa Rocha, Fernanda Rodrigues de Almeida, Pedro Ferrari Sales da Cunha, Stella Cristina Silva Souza, Gabriel Prado Saad, Thiago Adriano de Deus Queiroz Santos, Ariela Mota Ferreira, Timothy C. Tan, Maria Carmo Pereira Nunes

**Affiliations:** 1 Postgraduate Course of Infectious Diseases and Tropical Medicine, School of Medicine, Universidade Federal de Minas Gerais, Belo Horizonte, MG, Brazil; 2 School of Medicine, Universidade Federal de Minas Gerais, Belo Horizonte, MG, Brazil; 3 Postgraduate Program in Health Sciences, State University of Montes Claros (Universidade Estadual de Montes Claros), Montes Claros, Minas Gerais, Brazil; 4 Department of Cardiology, Blacktown Hospital, University of Western Sydney, Sydney, New South Wales, Australia; Faculty of Medical Science - State University of Campinas, BRAZIL

## Abstract

**Background:**

Chagas cardiomyopathy (CDC) is associated with a poor prognosis compared to other cardiomyopathies. Speckle tracking echocardiography (STE), which provides direct assessment of myocardial fiber deformation, may be useful in predicting prognosis.

**Objective:**

This study assessed STE in CDC and compared with idiopathic cardiomyopathy (IDC), and also examined the incremental prognostic information of STE over left ventricular ejection fraction (LVEF) in these patients.

**Methods:**

We enrolled 112 patients, age of 56.7 ± 11.8 years, 81 with CDC and 31 with IDC. STE indices were obtained at baseline in all patients. The endpoint was a composite of death, hospitalization for heart failure, or need for heart transplantation.

**Results:**

Patients with IDC had worse LV systolic function compared to CDC, with LVEF of 34.5% vs 41.3%, p = 0.004, respectively. After adjustment for LVEF, there were no differences in STE values between CDC and IDC. During a median follow-up of 18.2 months (range, 11 to 22), 26 patients met the composite end point (24%). LV longitudinal strain was a strong predictor of adverse events, incremental to LVEF and E/e' ratio (HR 1.463, 95% CI 1.130–1.894; p = 0.004). The risk of cardiac events increased significantly in patients with GLS > - 12% (log-rank p = 0.035).

**Conclusions:**

STE indices were abnormal in patients with dilated cardiomyopathy, without differences between CDC and IDC. LV longitudinal strain was a powerful predictor of outcome, adding prognostic information beyond that provided by LVEF and E/e' ratio.

## Introduction

Chagas disease continues to be one of the most prevalent infectious diseases in Latin America, and has become a public health concern in non-endemic countries [1, 2]. Chagas cardiomyopathy constitutes the most serious manifestation of the disease, as a consequence of chronic myocarditis that leads to myocardial dysfunction and conduction-system defects [3, 4]. Chagas cardiomyopathy is associated with poor prognosis with higher mortality rates compared with other causes of heart failure [5–8]. A previous study showed that the patients with Chagas dilated cardiomyopathy (CDC) had worse survival than those with idiopathic dilated cardiomyopathy (IDC), independent of well-established prognostic of outcome in heart failure [8].

The structural derangement associated with intense collagen deposition within the left ventricle may play a role in determining the prognosis in Chagas cardiomyopathy [9]. Indeed, previous studies with cardiac magnetic resonance (CMR) have demonstrated that myocardial fibrosis is associated with impairment of systolic function, abnormal remodeling, and increased ventricular stiffness [10, 11]. Recently, a study demonstrated that left ventricular (LV) fibrosis assessed by CMR is independently related to major advserse cardiac events in patients with Chagas disease [12]

The pattern of myocardial fibrosis, however, varies according to the specific cause of cardiomyopathy[13]. Different types of myocardial fibrosis have been described according to the myopathic disease process [13]. In patients with Chagas disease, replacement myocardial fibrosis appears to be the predominant form, whereas in IDC diffuse interstitial fibrosis appears to be the major form [9, 10, 13].

Speckle tracking echocardiography (STE), a method that allows measurement of myocardial strain, has been utilized to demonstrate impaired cardiac function secondary to the degree of cardiac fibrosis [14]. However, the studies addressing the association of STE and myocardial fibrosis in Chagas disease have demonstrated controversial results [15, 16]. Gomes et al showed that patients with fibrosis on CMR imaging had lower global longitudinal, circumferential, and radial LVLV strain compared to controls without cardiac fibrosis despite similar LV ejection fraction [16]. Macedo et al also demonstrated a high correlation between longitudinal global speckle tracking strain with severity of myocardial fibrosis assessed by CMR in a cohort of 58 patients with different forms of chronic Chagas disease [15]. In their study, myocardial fibrosis directly correlated with LV ejection fraction. Longitudinal strain had no incremental role on the prediction of myocardial fibrosis, even in the patients with ventricular dysfunction [15].

Therefore, the present study was designed to assess differences in strain in patients with dilated cardiomyopathy due to Chagas disease versus idiopathic dilated cardiomyopathy and to characterize difference between idiopathic dilated cardiomyopathy with similar demographic characteristics. In addition, we aimed to determine the independent prognostic value of global longitudinal strain in the overall patient population with Chagas and non-Chagas dilated cardiomyopathy.

## Methods

### Study population

A total of 184 consecutive patients who were referred to our centre for clinical symptoms of heart failure due to Chagas or idiopathic cardiomyopathy between September 2015 to October 2016 were prospectively enrolled. The patients were considered eligible for the study if they had a diagnosis of Chagas dilated cardiomyopathy (CDC) as defined by LV enlargement with systolic function impairment due to Chagas disease, defined as LV ejection fraction below 50% assessed by 2D echocardiography, or controls i.e. idiopathic dilated cardiomyopathy (IDC) characterized by dilated LV with systolic dysfunction, and negative serological tests for Chagas disease in the absence of abnormal loading conditions or coronary artery disease sufficient to cause global systolic impairment [17].

Exclusion criteria included the presence of atrial fibrillation, and other cardiac or systemic diseases that may affect ventricular function, especially systemic hypertension, and rheumatic valve disease. The patients with symptoms suggestive of ischemic heart disease with risk factors for atherosclerosis underwent coronary angiography for rule out ischemic heart disease. Patients who had a pacemaker or an implanted cardioverter-defibrillator also were excluded.

Based on these inclusion and exclusion criteria, a final cohort of 112 was enrolled in the study. All patients were clinically stable on guideline based heart failure therapy. The study protocol was approved by the institutional review board.

### Echocardiographic evaluation

A standard comprehensive echocardiography was performed in all patients (Vivid Q, GE). All images were stored digitally and analysed off-line with EchoPac software (GE Medical). Quantification of cardiac chamber size and function were obtained in accordance with ASE guidelines [18]. The echocardiographic measurements were performed by one independent observer who was blinded to the etiology of the cardiomyopathy. Values from 3 consecutive cardiac cycles were measured and averaged.

LV diastolic function was assessed by pulsed-wave Doppler examination of mitral inflow and by tissue Doppler imaging (TDI). Peak systolic velocities (S’) as well as early diastolic (e’) and late diastolic (A’) velocities were measured at the medial and lateral border of the mitral annulus in the apical four-chamber view [19]. The E/e' ratio was then calculated using the average of the septal and lateral e’.

The continuous-wave Doppler tricuspid regurgitant velocity was used to estimate systolic pulmonary artery pressure (SPAP) using the simplified Bernoulli equation. RV function was also assessed using tissue Doppler imaging [20].

### Assessment of speckle tracking strain

**Fig 1:**
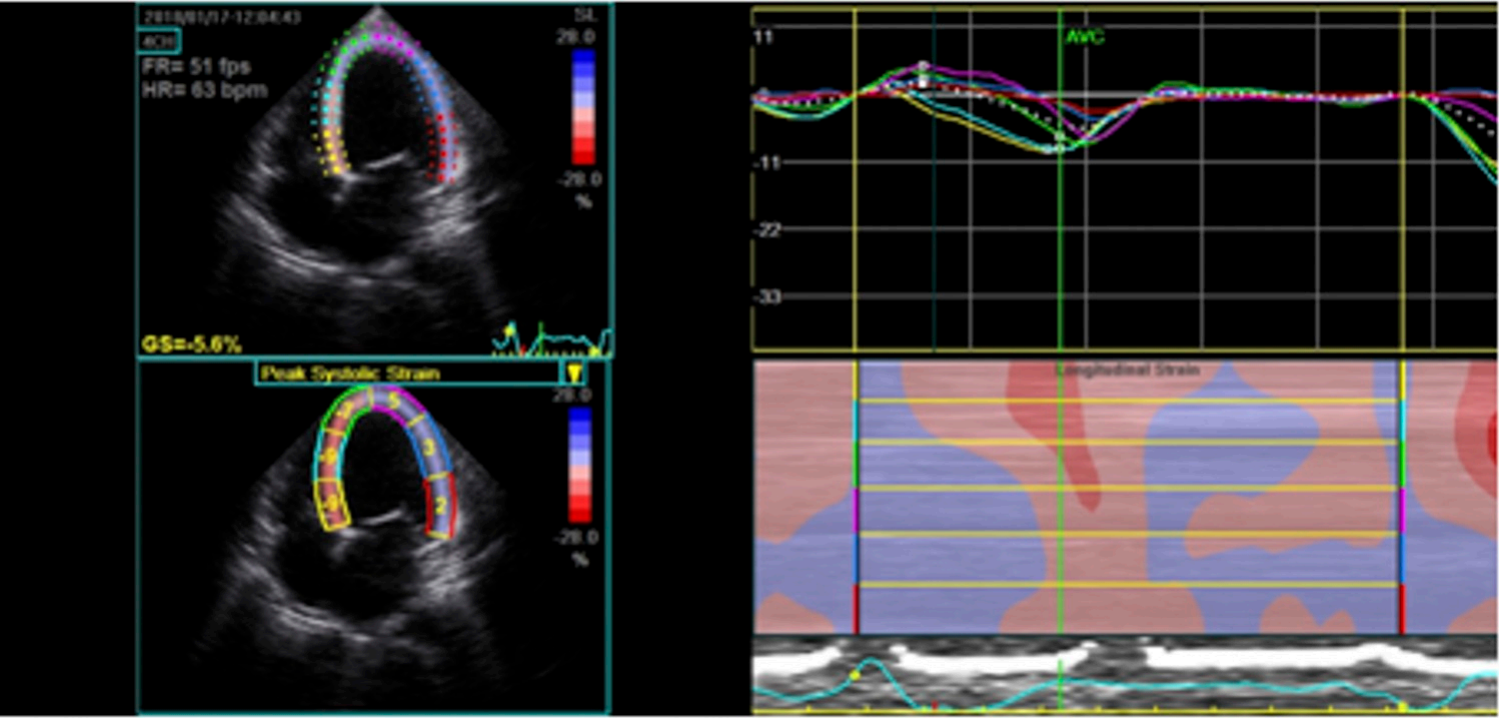
Peak systolic longitudinal strain of the left ventricle obtained at apical 4-chamber view. Parametric (color-coded) display of end-systolic strain (left upper panel); segmental peak systolic strain values are indicated (left lower panel); strain-time curves: colored curves displaying segmental strain (right upper panel); M-mode representation of peak systolic strain (right lower panel).

**Fig 2:**
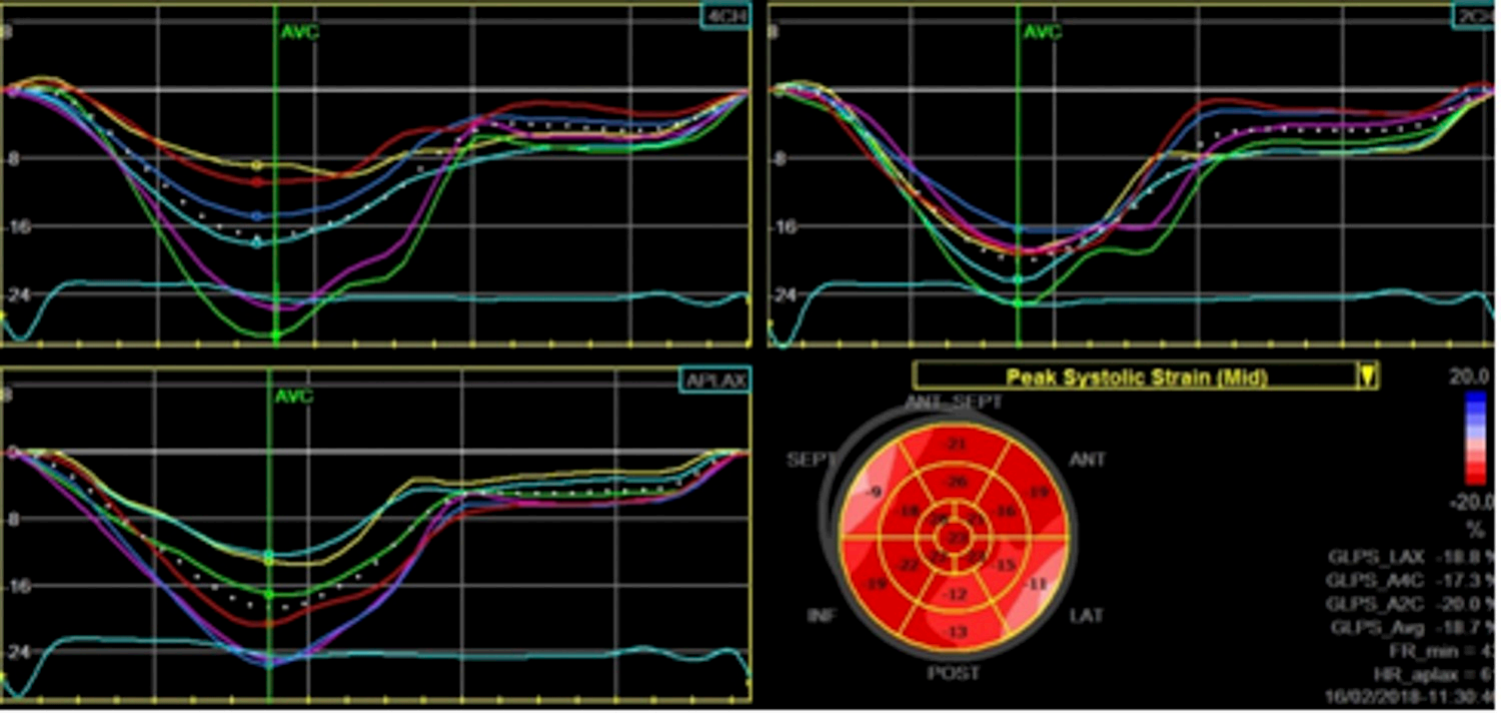
Left ventricular strain curves and bull’s eye plots. LV longitudinal strain curves derived from speckle-tracking analysis for each LV segment at apical 4-chamber view (left panel), paraesternal long-axis view (left lower panel), 2-chamber view (right upper panel), and bull’s eye” plot (right lower panel).

Images of the LV in the apical 4-, 2- and 3-chamber views were obtained for analysis, as previously described.[21] The LV was divided into 18 segments and, by using a dedicated software package (Echopac PC, Version 7.0.X, GE Healthcare, Fairfield, CT), 2D LV longitudinal strain was obtained for each segment[22]. Longitudinal systolic strain was determined from apical views (Fig 1). The value of global longitudinal strain was obtained by averaging all LV segments, which was used for comparisons with the IDC controls (Fig 2).

**Fig 3:**
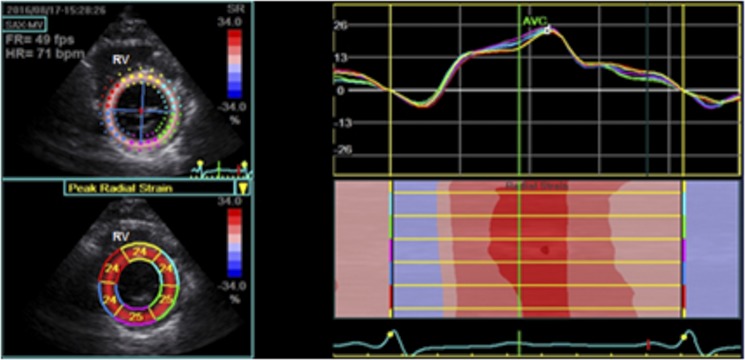
Radial strain of the left ventricle selected at the papillary muscles. The myocardium was divided into six segments (left upper panel); segmental strain graphically displayed (left lower panel); colored curves are outlined (right upper panel); M-mode color-coded display of peak systolic strain (right lower panel).

**Fig 4:**
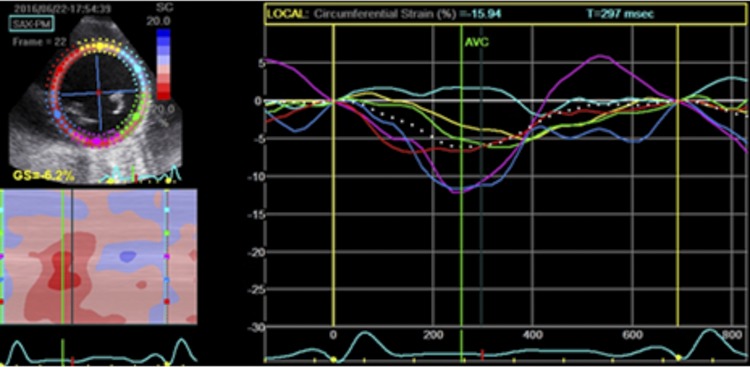
Left ventricular circumferential strain at the papillary muscles, with tracked of the six segments (left upper panel); M-mode representation of peak systolic strain (left lower panel); segmental end-systolic strain graphically displayed (left panel).

Radial (RS) and circumferential strain (CS) were obtained in all 18 segments of the three short-axis views. The average of peak systolic radial and circumferential strain values was calculated to derive the global LV radial and circumferential strain and strain rates (Figs 3 and 4). LV twist was calculated as the difference between apical and basal rotations at each corresponding time point.

RV strain was assessed from a dedicated apical focused four-chamber view with the sample areas placed in the basal, mid, and apical segments of the RV free wall and ventricular septum. A region of interest was manually drawn to include the entire myocardial wall and to exclude the pericardium. Patients in which >2 segments per ventricle revealed inadequate tracking despite attempts to readjust the region of interest position were excluded from speckle tracking strain analysis.

### Endpoint definition

The endpoint was a composite endpoint of death, heart failure hospitalization, or cardiac transplantation. Follow-up data were obtained during clinical follow-up appointment or telephone interviews. To satisfy the assumption of the independence of events, hospitalization for heart failure, during which cardiac transplant was recommended or patient died, was not defined as a separate endpoint.

### Statistical analysis

Categorical variables were presented as numbers and percentages, whereas continuous variables were expressed as mean ± standard deviation (SD). The measurements of patients with CDC and IDC were compared using Chi-square test, unpaired Student’s t-test or Mann-Whitney test, as appropriate. Multivariable Cox proportional hazards analysis was used to identify variables associated with adverse events, considering the time until the occurrence of an endpoint of interest.

The potential predictive variables of outcome included in the Cox analysis were NYHA functional class, LV ejection fraction, LA volume index, E/e' ratio, longitudinal RV strain, and global longitudinal strain. In order to prevent overfitting, the shrinkage factor of regression coefficients was applied after the model has been fitted by traditional methods [23].

All analyses were performed using SPSS 21.0 (SPSS Inc, Chicago, IL)

## Results

### Baseline clinical characteristics

Of the 112 patients recruited, 81 patients had CDC whereas 31 had IDC. The mean age was 56.7 ± 11.8 years, and 63 patients were men (56%). The baseline clinical characteristics of the study population, comparing Chagas with idiopathic cardiomyopathy, are summarized in Table 1. Most of the patients (72%) were in NYHA functional class II and 15 patients were class I at the time of inclusion in the study. One hundred and seven patients (96%) were on angiotensin-converting enzyme inhibitors or angiotensin receptor blockers therapy, 77 (69%) beta-blockers, 68 (61%) diuretics, and 21 (19%) anticoagulants.

**Table 1:** Clinical features of the patients stratified according to underlying cause of cardiomyopathy.

Variable*		Chagas cardiomyopathy (n = 81)	Idiopathic cardiomyopathy (n = 31)	P value
Age (years)		56.9 ± 10.5	56.1 ± 4.8	0.752
Male (n/%)		48 (59)	15 (48)	0.299
Body surface area (m^2^)		1.72 ± 0.2	1.79 ± 0.2	0.091
NYHA class	I	12 (15)	3 (10)	0.021
	II-III	69 (85)	28 (90)	
Right ventricular failure^†^		9 (11)	9 (29)	0.021
Heart rate (bpm)		69.3 ± 13.7	71.4 ± 10.5	0.430
SBP (mmHg)		117.9 ± 15.7	113.2 ± 13.7	0.145
DBP (mmHg)		77.2 ± 11.1	72.1 ± 9.5	0.025
**Medications**				
ACE inhibitors		35 (43)	19 (61)	0.096
Diuretics		43 (53)	25 (81)	0.009
Beta-blockers		51 (63)	26 (84)	0.041
Amiodarone		21 (26)	3 (10)	0.073
Anticoagulants		15 (19)	6 (19)	0.557
**Echocardiographic parameters**				
LVDd (mm)		59.3 ± 9.3	62.5 ± 11.9	0.135
LVSd (mm)		46.9 ± 9.9	52.2 ± 10.9	0.016
LVEF (%)		41.25 ± 9.05	34.48 ± 11.18	0.004
LA volume index (mL/m^2^)		36.4 ± 15.0	41.1 ± 19.1	0.175
E (cm/s)		68.24 ± 27.15	73.52 ± 28.62	0.381
A (cm/s)		74.88 ± 21.34	71.39 ± 26.09	0.510
DT (ms)		218.10 ± 77.15	186.26 ± 90.46	0.057
E/e’ ratio		12.34 ± 5.91	14.99 ± 8.12	0.106
PASP (mmHg)		33.5 ± 27.3	37.1 ± 12.3	0.492
Peak systolic velocity (cm/s)††		11.6 ± 2.9	11.4 ± 3.3	0.735

*Variables are expressed as mean value ± SD or number (percentage) of patients.

†: Manifestations of right-sided heart failure.

††Peak systolic velocity at the tricuspid annulus

A = late diastolic transmitral flow velocity; BSA = body surface area; DBP = diastolic blood pressure; DT = deceleration time; E = early diastolic transmitral flow velocity; e’ = early diastolic mitral annular velocity at septal and lateral mitral annulus, E/e’ = ratio of the early diastolic transmitral flow velocity to early diastolic mitral annular velocity (average at septal and lateral mitral annulus); LA = left atrial; LVDd = left ventricular end-diastolic diameter; LVEF = left ventricular ejection fraction; LVSd = left ventricular end-systolic diameter; NYHA = New York Heart Association; PASP = pulmonary artery systolic pressure; RV = right ventricular; RVEF = right ventricular ejection fraction; SBP = systolic blood pressure; TR = tricuspid regurgitation.

The demographic features were similar between the groups. However, patients with IDC presented more frequently in class III with evidence of systemic congestion than patients with CDC. Similarly, diuretics use was more frequent in IDC, which also reflects the heart failure severity.

Right bundle-branch block was more frequent in the patients with Chagas than in those with IDC (57% vs 10%; p <0.001). The proportion of patients with premature ventricular contractions was similar between the groups. No difference was observed in regard to other ECG variables.

### Echocardiographic measurements

Echocardiographic parameters to measure LV function according to the types of cardiomyopathy are shown on Table 1. Patients with IDC had worse LV systolic function compared to CDC, with ejection fraction of 34.5% vs 41.3%, p = 0.004, respectively. There was no difference in the indexes of LV diastolic function in patients with CDC and IDC. Additionally, RV function was similar between the patients, except RV end-diastolic volume that was higher in IDC than CDC (Table 1). LV apical aneurysm was found in 14 patients (17%) with thrombi in 5 patients (6%), only in patients with CDC. Segmental wall motion abnormalities were detected in 40 patients (49%) with CDC and in 3 (10%) with IDC, being more frequent in inferolateral and inferior walls.

### Speckle tracking echocardiographic strain

Global LV longitudinal strain was lower in patients with IDC than CDC (10.5 ± 4.4 vs 12.5 ± 4.2; p = 0.032, respectively), especially in the inferoseptal and anteroseptal walls (Table 2). Circumferential strain was reduced only in anteroseptal wall in patients with ICD compared to CDC, with no difference in the global circumferential and radial strain. Similarly, RV longitudinal strain did not differ between groups.

**Table 2:** Ventricular speckle tracking strain of patients with Chagas cardiomyopathy compared to idiopathic cardiomyopathy.

Walls*	Chagas cardiomyopathy (n = 81)	Idiopathic cardiomyopathy (n = 31)	P value
**LV Longitudinal strain (%)**			
Antero-septal	12.07 ± 5.02	9.01 ± 5.16	0.005
Anterior	12.55 ± 4.43	10.61 ± 5.01	0.064
Antero-lateral	11.85 ± 5.35	10.66 ± 5.26	0.295
Infero-lateral	11.14 ± 5.73	11.20 ± 4.98	0.956
Inferior	13.12 ± 5.34	10.59 ± 5.59	0.024
Infero-septal	14.34 ± 5.09	9.72 ± 5.69	<0.001
Global strain	12.46 ± 4.22	10.49 ± 4.43	0.032
Strain rate (s^-1^)	0.73 ± 0.23	0.71 ± 0.28	0.672
**LV Radial strain (%)**			
Antero-septal	14.76 ± 7.51	14.03 ± 9.49	0.701
Anterior	15.51 ± 8.21	16.44 ± 9.71	0.615
Antero-lateral	16.28 ± 8.18	18.34 ± 9.25	0.254
Infero-lateral	16.35 ± 8.56	18.70 ± 9.29	0.206
Inferior	16.28 ± 8.66	17.17 ± 9.34	0.636
Infero-septal	15.72 ± 8.30	15.00 ± 9.34	0.691
Global strain	15.82 ± 7.83	16.61 ± 9.14	0.653
Strain rate (s^-1^)	0.73 ± 0.23	0.71 ± 0,28	0.672
Twist	7.40 ± 4.23	8.85 ± 5.60	0.200
**LV Circumferential strain (%)**			
Antero-septal	14.49 ± 6.32	11.65 ± 5.76	0.027
Anterior	12.61 ± 5.57	10.78 ± 4.35	0.072
Antero-lateral	10.36 ± 4.30	11.40 ± 3.74	0.216
Infero-lateral	10.15 ± 4.29	11.84 ± 4.55	0.069
Inferior	10.54 ± 4.95	10.86 ± 3.99	0.721
Infero-septal	14.84 ± 7.20	12.26 ± 5.70	0.052
Global strain	10.38 ± 4.05	9.30 ± 3.77	0.202
Strain rate (s^-1^)	0.96 ± 0.27	1.02 ± 0.32	0.375
**RV Longitudinal Strain (%)**			
Septal wall	11.5 ± 4.0	10.1 ± 4.9	0.135
RV free wall	18.5 ± 7.1	15.8 ± 5.7	0.064
Global RV strain	14.9 ± 4.8	12.9 ± 4.9	0.052
Strain rate (s^-1^)	1.02 ± 0.34	0.96 ± 0.34	0.414

*The table presents absolute values of strain.

LV = left ventricular; RV = right ventricular

Since IDC patients had lower LV ejection fraction than CDC, a sub-analysis was performed selecting patients with similar degree of LV systolic dysfunction (LV ejection fraction ≤ 35%; 46 patients). The results showed no differences in global longitudinal (7.6 ± 2.8 vs 7.8 ± 1.9; p = 0.721), radial (12.3 ± 7.2 vs 11.0 ± 5.2; p = 0.523) or circumferential (7.2 ± 3.2 vs 6.5 ± 3.6; p = 0.567) strain in patients with IDC compared with CDC, respectively. Similarly, RV longitudinal strain was 11.3 ± 5.1 in IDC and 12.9 ± 4.9 in CDC (p = 0.331).

### Predictors of outcome

During a median follow-up of 18.2 months (range, 11 to 22), 26 events occurred (14 heart failure hospitalization, 8 cardiac deaths, and 4 cardiac transplant). Of patients with idiopathic cardiomyopathy, 6 events (19%) occurred compared with 20 (25%) in Chagas cardiomyopathy (P = 0.508).

By univariate analysis by Cox model, several variables were associated with poor outcome (Table 3). In the multivariate analysis including the entire cohort, the independent predictors of adverse events were LV ejection fraction (Hazard ratio [HR] 0.839; 95% confidence interval [CI] 0.760–0.925; p <0.001), E/e' ratio (HR 1.060; 95% CI 1.015–1.108; p = 0.008), and LV longitudinal strain (HR 1.365; 95% CI 1.106–1.686; p = 0.004) (Table 4).

**Table 3:** Predictors of adverse cardiac events in patients with dilated cardiomyopathy: Univariate analysis.

Variable	Without events (n = 86)	With events (n = 26)	HR (95% CI)	P
Age (years)	55.9 ± 11.7	58.6 ± 21.1	1.004 (0.968–1.042)	0.824
Male gender (n/%)	53 (62)	10 (38)	0.413 (0.182–0.935)	0.034
NYHA class III/IV (n/%)	9 (10)	7 (27)	1.618 (0.635–4.119)	0.313
LV ejection fraction (%)	41.8 ± 9.3	33.2 ± 8.8	0.936 (0.900–0.973)	0.001
E/e' ratio	9.4 ± 4.9	14.9 ± 6.1	1.088 (1.036–1.143)	0.001
LA volume index (mL/m^2^)	35.4 ± 13.2	50.9 ± 20.9	1.030 (1.012–1.048)	0.001
RV systolic velocity (cm/s)*	11.9 ± 2.9	10.4 ± 2.9	0.866 (0.738–1.016)	0.077
Speckle tracking strain				
Longitudinal strain (%)	12.7 ± 4.3	10.2 ± 3.9	0.879 (0.784–0.985)	0.026
Radial strain (%)	17.4 ± 8.5	12.3 ± 6.4	0.917 (0.856–0.982)	0.013
Circumferential (%)	10.6 ± 3.8	8.9 ± 4.3	0.915 (0.814–1.028)	0.134
Longitudinal RV strain (%)	15.1 ± 4.9	12.9 ± 4.3	0.935 (0.848–1.032)	0.185

*Peak systolic velocity at the tricuspid annulus

HR = hazard ratio, LA = left atrial; LV = left ventricular; NYHA = New York Heart Association; RV = right ventricular

**Table 4:** Multivariable Cox proportional-hazards analysis for predicting adverse cardiac events in the overall patient population with dilated cardiomyopathy.

Variables*	Hazard ratio^†^	95% CI	Z value	P value
LV ejection fraction (%)	0.839	0.760–0.925	-3.532	0.0004
E/e' ratio	1.060	1.015–1.108	2.638	0.0083
LV global longitudinal strain (%)	1.365	1.106–1.686	2.892	0.0038

* Fitted model estimates corrected by shrinkage factor of 0.8139

† Shrinkage hazard ratio

The AUC increased slightly with the inclusion of LV strain in the model, from 0.818 (95% CI 0.732–0.905) to 0.839 (95% CI 0.757–0.921).

**Fig 5:**
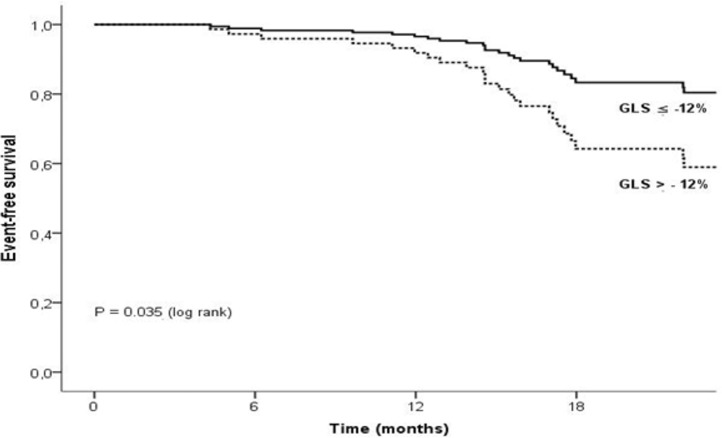
Event-free survival rate according to left ventricular global longitudinal strain (LVGLS) cut-off. Kaplan–Meier time curves stratified according to an LVGLS cut-off of –12%. Those with an LVGLS ≤ -12% were significantly more likely to be free of the event compared with those with an LVGLS of > - 12% or more.

The risk of cardiac events increased significantly in patients with GLS > - 12% (log-rank p = 0.035) (Fig 5).

## Discussion

This study evaluated the utility of strain in patients with dilated cardiomyopathy and sought to identify differences in strain pattern and values according to the underlying cause of the cardiomyopathy. Speckle tracking echocardiographic deformation indices are abnormal in patients in patients with dilated cardiomyopathy. Specifically, myocardial contractility by STE in patients with CDC does not appear to be more impaired compared with IDC with similar LV ejection fraction. Global LV longitudinal strain is associated with cardiac events in patients with dilated cardiomyopathy, regardless of the etiology of the cardiomyopathy, independent of other parameters of poor prognosis including LV ejection fraction and E/e' ratio.

Different types of strain have been used to assess myocardial deformation. LV longitudinal strain reflects ventricular long axis function, controlled predominantly by subendocardial fibers [24]. A previous study with Chagas disease found that radial and circumferential strain could be more accurate for early detection of myocardial involvement than longitudinal strain [25]. Furthermore, when the LV becomes enlarged and more spherical in dilated cardiomyopathy, the contribution from circumferential shortening will increase further relative to longitudinal shortening. Radial strain results from the combination of longitudinal and circumferential fibre shortening and, therefore, may be a better measure of myocardial deformation alone or in combination [24].

### Speckle tracking echocardiography in Chagas disease

Myocardial deformation imaging with echocardiography can be measured using tissue Doppler imaging and 2- and 3-dimensional speckle tracking echocardiography [14, 22]. Tissue Doppler-derived strain has a number of limitations, and strain measurements based on speckle tracking have become a method of choice to assess myocardial deformation.

In the setting of Chagas disease, the traditional assessment of regional contractile function relies on a qualitative visual estimation of regional thickening and contraction. Speckle tracking echocardiography has allowed for the increasing recognition of subclinical myocardial dysfunction, particularly in patients in the indeterminate form of Chagas disease [15, 25–28]. A previous study in asymptomatic patients with Chagas disease with normal ECG demonstrated reduced LV strain compared to controls, especially global radial strain at the basal segments of inferior, inferoseptal, and inferolatateral walls [27]. Similarly, another study with patients in indeterminate form of Chagas disease had lower global radial strain in the inferior segments compared to controls [25]. In contrast, Gomes et al [16] including 125 Chagas disease patients found no differences in global longitudinal, circumferential, and radial LV speckle-tracking strain among controls, patients in the indeterminate form, and asymptomatic patients with isolated changes on the ECG. However, patients who had myocardial fibrosis on cardiac magnetic resonance imaging presented with lower global longitudinal, circumferential and radial speckle tracking strain than those without cardiac fibrosis despite similar LV systolic function by ejection fraction [16]. In another study including patients in different forms of chronic Chagas disease a high correlation between longitudinal global speckle tracking strain with myocardial fibrosis was found. [15] Nevertheless, after adjustment for LV ejection fraction, speckle tracking strain was not associated with myocardial fibrosis [15].

Different from previous studies, the present study enrolled only Chagas disease patients with dilated cardiomyopathy and systolic dysfunction and compared it to a control population with non-Chagas dilated cardiomyopathy, defined by negative anti-*T*.*cruzi* serology. A homogenous population was selected to search for differences in the pattern of myocardial strain potentially related to myocardial fibrosis. The finding of reduced radial strain despite higher LV ejection fraction in CDC supports the concept that radial strain appears to be more accurate to detect myocardial dysfunction than longitudinal strain in the setting of Chagas disease.

Regueiro et al [10] reported that in CDC, 52% of the patients had myocardial fibrosis and/or necrosis, with heterogeneous pattern of involvement including subendocardial, midwall, subepicardial, and transmural. Since radial thickening is determined by both circumferential and longitudinal myocardial fibre shortening, it expresses better the overall ventricular function in CDC.

### Prognostic value of LV longitudinal strain

While strain by speckle-tracking has been shown to have clinical utility in the identification of systolic dysfunction in the context of normal ejection fraction, there is also a growing body of evidence to indicate that strain may also have prognostic value in patients with heart failure[29–31]. Ejection fraction, derived from changes in ventricular volumes, does not necessarily reflect myocardial muscle or sarcomeric shortening. As such myocardial strain and ejection fraction (which is more of a measure of global function) could be seen as distinct entities [32]

Although myocardial strain is a comprehensive indicator of LV function, few data are available regarding its prognostic implications in Chagas disease. Previous studies have examined the LV strain as a potential predictor for Chagas disease progression [27, 28]. In a study including a wide spectrum of clinical presentation of patients with Chagas disease, global LV longitudinal and radial strain progressively decreased along with increased of brain natriuretic peptide levels as diastolic dysfunction severity increased [33]. In our study, we assessed the association between LV global longitudinal strain and the occurrence of cardiovascular events, regardless of LVEF and diastolic filling pressures, which are well-established prognostic markers in dilated cardiomyopathy. Our results suggest that measurement of LV global longitudinal strain can improve risk stratification in these patients and may contribute to better decision making for further treatment in this population.

### Study limitations

Patients with Chagas cardiomyopathy were referral for assessment of heart disease at the time of positive serological tests whereas those with IDC had the diagnosis after the onset of symptoms. It may explain the more severe clinical presentation of IDC than CDC. The small number of events limited a stratified analysis according to the etiology of the cardiomyopathy.

Although strain is considered relative load independent, systolic strain is largely an ejection phase index, which is substantially dependent on preload and afterload. Additionally, cardiac magnetic resonance to assess myocardial fibrosis was not performed in our study.

## Conclusions

Speckle tracking echocardiographic deformation indices were abnormal in patients with dilated cardiomyopathy. Patients with Chagas cardiomyopathy presented similar STE values with idiopathic cardiomyopathy, after adjustment for the degree of LV systolic dysfunction. LV longitudinal strain was a powerful predictor of cardiac events in patients with dilated cardiomyopathy, which provides prognostic information beyond to LV ejection fraction and E/e' ratio.
